# 3D printing in rheumatology

**DOI:** 10.1016/j.ero.2026.100202

**Published:** 2026-06-16

**Authors:** Samuel Guigo, Sophie Débonnaire, Zoé Rapin, Alain Saraux

**Affiliations:** 1 W.PRINT, Clinical Research and Innovation Department, Centre Hospitalier Universitaire Brest, Brest, France; 2 Université de Bretagne Occidentale (Univ Brest), Department of Rheumatology and pole Pharmacie Recherche Santé Publique et Santé au Travail, Centre Hospitalier Universitaire Brest, Institut National de la Santé et de la Recherche Medicale Lymphocytes B, Autoimmunité, Immunothérapies (U1227), LabEx Immunotherapy, Graft, Oncology, Brest, France

## Abstract

Three-dimensional (3D) printing, or additive manufacturing, revolutionises medicine by creating customised objects from digital models, layer by layer. In hospitals, technologies like Fused Deposition Modelling (FDM), stereolithography, and laser powder bed fusion are used, each offering unique advantages. Materials range from biocompatible polymers for orthoses to metal alloys for implants. FDM printers are affordable and suitable for prototyping but lack precision. Stereolithography provides high resolution and biocompatibility, ideal for medical devices, though postprocessing is required. PolyJet technology produces high-quality, multicoloured parts but is costly, limiting its use to specialised settings. Applications include anatomical models for preoperative planning, surgical guides, custom orthoses, and implants, enhancing precision and patient outcomes. In education, 3D-printed models aid in training for complex procedures. Research explores regenerative medicine, such as scaffolds for tissue regeneration and drug delivery systems.

In rheumatology, 3D printing shows potential for custom orthoses, educational tools, and bioprinting cartilage. However, adoption is limited by methodological, economic, and environmental barriers. Waste production, material strength, and cost remain challenges. Evidence is mostly observational, with few randomised trials. Future prospects include biocompatible materials, improved segmentation techniques, and smartphone-integrated modelling tools. Custom prostheses and 3D-printed medications offer personalised care but require rigorous safety and regulatory frameworks. In conclusion, although 3D printing holds significant promise for rheumatology and the broader medical field, its integration into clinical practice depends on stronger evidence, cost-effectiveness, and sustainable practices. Its development could be advanced through academic initiatives as well as through collaborations with 3D printing firms to conduct formal clinical trials.

Three-dimensional (3D) printing, or additive manufacturing, is an innovative technology that enables the production of customised objects from digital models acquired through 3D imaging (primarily computed tomography [CT] scans) by successively adding material layer by layer. In medicine, it contributes to the evolution of traditional clinical practices by offering tailored solutions for care, education, and research. In hospital settings, various 3D printing technologies are used, ranging from thermoplastic filament deposition processes (Fused Deposition Modelling [FDM]) to stereolithography or laser powder bed fusion techniques, each offering specific advantages depending on the clinical indication. The materials used cover a wide range, from biocompatible polymers intended for external orthoses and anatomical models to metal alloys used for the fabrication of structural implants.

The different 3D printing technologies have distinct characteristics.

○ **Filament printers (FDM)** constitute an accessible solution suitable for rapid prototyping and educational uses. However, their slow execution and limited production capacity restrict their use for devices requiring high precision. The filaments used (approximately 20€ per kilogram) are primarily polylactic acid, which is biodegradable and bio-based but brittle, and acrylonitrile butadiene styrene, which is more resistant but requires an enclosed printer and high temperatures.
○ **Vat photopolymerisation (stereolithography)**, which uses liquid resin, offers better resolution and interesting properties for the medical field, particularly biocompatibility, sterilizability, and a certain flexibility of the final product, at an intermediate cost. However, it involves postprocessing constraints, such as support removal and alcohol rinsing.
○ **PolyJet technology**, which uses liquid resins sprayed through multiple nozzles, allows for the production of multicoloured, biocompatible, and high-quality parts with water-soluble supports, but its high cost and technical constraints make it primarily suitable for professional or specialised hospital use. PolyJet resins are more expensive, and the specific ones can reach 400€ per litre per colour, which explains their mainly professional use.

Thus, the choice of technology depends on the desired compromise between cost, precision, mechanical properties, and clinical requirements. The 3D printer offers an interesting level of resolution with filament layers ranging from 0.4 mm to 0.2 mm, resulting in 3D-printed parts of good precision. Initially, solid filaments are liquefied by the printer nozzle and then solidified upon contact with the base.

The applications are numerous and include the design of anatomical models, surgical guides, custom orthoses, implantable prostheses, and, more recently, individualised pharmaceutical devices.

Over the past few years, the integration of 3D printing within hospital facilities has accelerated, as seen with platforms like W.*PRINT* in our hospital, which leverages this technology for patient care, teaching, and research. However, rheumatology has yet to widely adopt this innovation, prompting questions about its application in other medical specialties and its relevance for rheumatologists. Our aim is to share our perspective, drawing from the experience of W.*PRINT*—one of the first 3D printing platforms established in a French hospital.

## APPLICATIONS OF 3D PRINTING IN DIFFERENT MEDICAL AND SURGICAL SPECIALTIES

3D printing first allows, in the context of care, the creation of patient-specific anatomical models from imaging data (Table [[1], [2], [3], [4]], Fig). These models improve spatial understanding of anatomy and facilitate preoperative planning as well as the simulation of technical procedures. In cardiology [5], for example, these models contribute to better anticipation of complex procedures and optimisation of interventional strategies [6]. In orthopaedic surgery [7], in the context of tibial plateau fractures, the use of 3D-printed models for surgical planning appears promising. It is significantly associated with a reduction in intraoperative complications and an improvement in postoperative recovery. Additionally, 3D printing allows the fabrication of customised medical devices, including surgical guides, implants, dental prostheses [8], and support devices adapted to each patient’s specific anatomy. This customisation improves the precision of medical procedures, the fit of devices, and functional outcomes. It proves particularly useful in complex or atypical anatomical situations [9], for which standardised solutions are insufficient or even ineffective.

**Table:** Current use of 3D printers in the medical field in general and in rheumatology

	Current use	Expected benefits
***In general***		
Care	- Preoperative planning: Patient-specific anatomical models derived from medical imaging, used for planning interventions and simulating technical procedures - Simulation: Custom fabrication of surgical guides, implants, prostheses, and support devices adapted to the patient’s anatomy - Customisation of medical devices	- Better anatomical understanding - Optimisation of surgical procedures - Reduction of intraoperative complications - Improvement of postoperative recovery - Increased precision of medical procedures - Better fit of devices - Improvement of functional outcomes and patient comfort
Education	- Training and anatomical education: Use of 3D-printed models for learning surgical procedures and teaching anatomy, particularly in rare clinical situations (improved skill acquisition)	- Standardisation of training - Reduced dependence on real clinical exposure
Research	- Therapeutic applications and tissue engineering: Design of 3D-printed biomedical scaffolds for tissue regeneration and postoperative management	- Stimulation of angiogenesis - Improved healing - Support for tissue repair and management of pathological residues
***In rheumatology***		
Care	- Little or no use	- Custom orthoses: pain relief indication
Education	- Little or no use	- Visual therapeutic education of patients with 3D models of their pathologies (narrow lumbar canal, osteoarthritis) - Training models for ultrasound-guided joint injections with gelatin-based support and anatomical models for lumbar puncture on deformed spine
Research	- Development of adaptive hybrid constructions produced with multimaterial [1] - Hybrid bioactive scaffold from pearl powder for bone regeneration (tested in rats) [2] - Multiphase scaffold [3] - Porous scaffold based on fish skin gelatin (indication: osteoarthritis) tested in rats	- Creation of implantable cartilage for osteoarthritis treatment - Regeneration of annulus fibrosus in acute low back pain [4] - Development of combined 3D-printed medications (corticosteroids, NSAIDs, analgesics) - Link with 3D modelling for quantification of joint volumes during rheumatoid arthritis flares

3D, 3 dimensional; NSAIDs, Non Steroidal Antiinflammatory Drugs.

**Figure:**
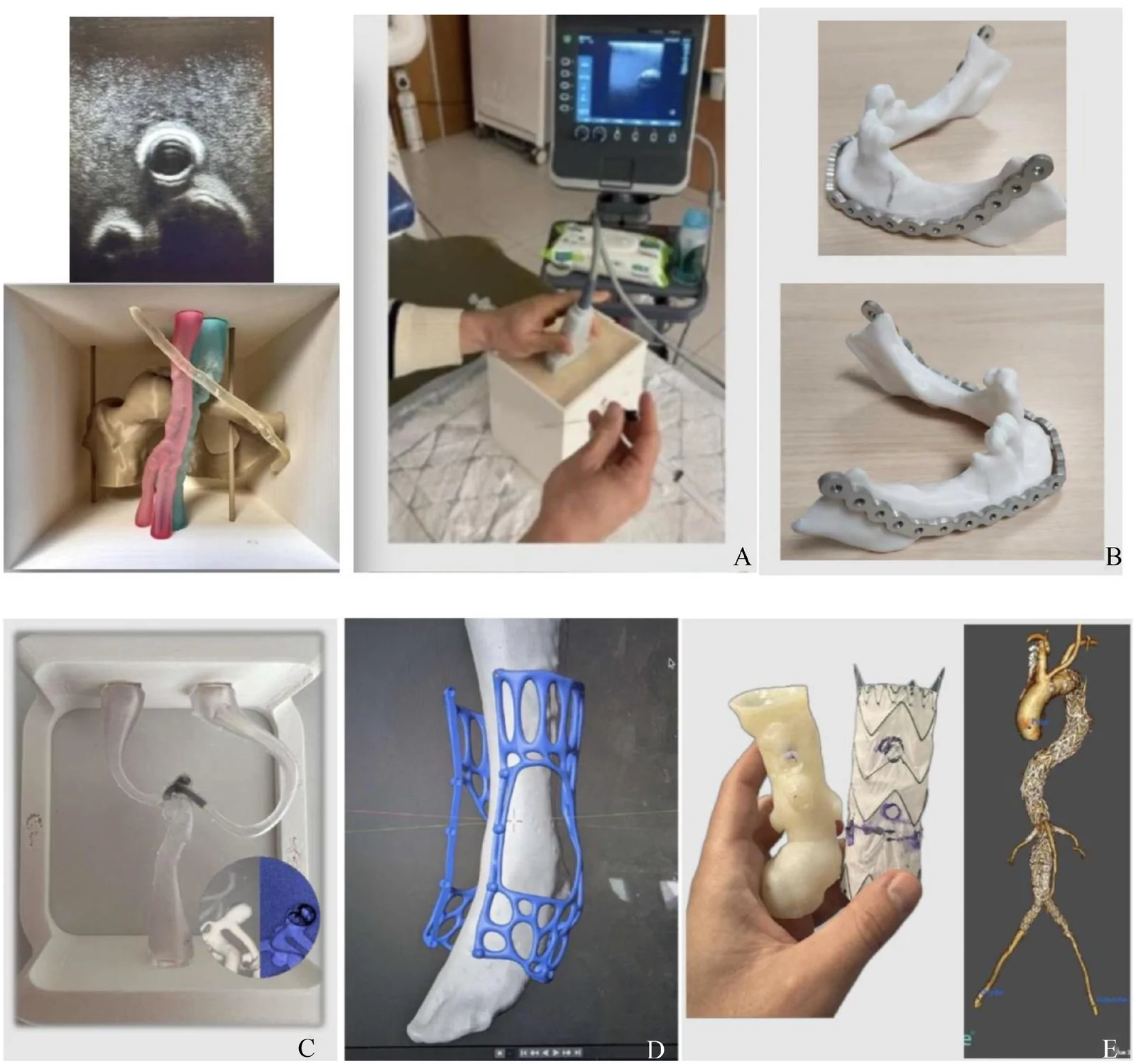
Example of 3 dimensional (3D) printing. (A) Anatomical model of femoral puncture under ultrasound, PolyJet technology, echogenic environment. (B) Custom anatomical model of mandible for ORL surgery. (C) Training model for carotido-styloid aneurysm, Anev3D. (D) 3D-printed orthosis, Skelenn company. (E) Custom anatomical model for vascular surgery. CHU, Centre Hospitalier Universitaire; ORL, Otorhinolaryngology. Photo by Samuel Guigo, W.PRINT, CHU Brest, France.

3D printing is also a useful educational tool for training healthcare professionals, offering realistic, reproducible, and manipulable anatomical supports. Literature reviews in neurosurgery and otorhinolaryngology [10] highlight the interest of 3D-printed models for learning complex anatomy and training in surgical procedures, as a complement or alternative to traditional methods. In gynaecology-obstetrics, particularly in the context of fetal surgery [11], 3D printing makes it possible to compensate for the rarity of malformations and limited access to practice by providing simulation models adapted to the training of specialised teams. An in-house 3D-printed simulator could even significantly outperform the commercial model [12].

Finally, emerging research applications position 3D printing in the field of regenerative medicine. The design of 3D-printed biomedical scaffolds supports tissue regeneration, promotes angiogenesis, and improves postoperative healing [1]. These devices can also contribute to the management of pathological residues after surgical excision, opening innovative therapeutic perspectives in addition to conventional treatments.

## APPLICATIONS OF 3D PRINTING IN RHEUMATOLOGY

Rheumatology is a specialty where 3D printing holds promise. Studies in this field, though few, are currently at the in vitro research stage and sometimes in vivo in animals, primarily highlighting the creation of scaffolds to promote bone regeneration.

3D printing could, however, open new perspectives in rheumatology (Table [[1], [2], [3], [4]]), with applications in clinical care, education, and research.

• In clinical practice, it could enable the design of custom orthoses for pain relief, develop therapeutic education tools based on personalised anatomical models, or facilitate patient positioning during imaging examinations. However, under good clinical practice or regulatory requirements, these devices are generally manufactured by external partners, and this fact currently limits the routine implementation. In education, the use of 3D-printed models could improve the learning of technical procedures, such as ultrasound-guided joint injections (interface made with gelatin and soy to make it echogenic) or lumbar punctures in complex anatomical contexts. Nevertheless, although biomaterials and tissue engineering using 3D printing are already widely used, it remains technically challenging to produce realistic soft-tissue layers. So, only low-cost production should be in-house by most centres.
• It could also be used for patient awareness of structural damage [2] or integrated in a multimodal teaching concept for medical students [3]. In research, this technology could foster innovations such as the bioprinting of cartilage tissues, with research growing since the 2000s [4,[13], [14], [15], [16], [17]], the printing of personalised medications, or even the 3D quantification of joint volumes, which could contribute to a better assessment of inflammatory activity, particularly in rheumatoid arthritis.

## CURRENT LIMITATIONS AND FUTURE OUTLOOK

Nevertheless, its integration into clinical practice remains limited by several methodological, economic, and environmental barriers.

The production of waste related to 3D printing processes, including printing supports and manufacturing failures, as well as the potential emission of nanoplastics, raises environmental concerns that are still little explored in the medical literature. These aspects will need to be taken into account in the overall assessment of the benefit-risk ratio of these technologies.

The study of material selection has already seen progress with the knowledge of each material’s resistance characteristics. However, it can be complex to find a material strong enough to support significant weight, particularly in certain lower limb aids or orthoses [18].

The current level of evidence remains generally low to moderate. The available data are mostly based on case series and observational studies, with a limited number of randomised controlled trials. In rheumatology, although custom 3D-printed orthoses and guides seem to improve morphological adaptation and patient satisfaction [19], their long-term clinical impact and superiority over conventional devices remain to be demonstrated.

Cost is a major obstacle to the widespread adoption of 3D printing. It varies depending on the complexity of the device (orthosis, knee, or spinal joint model), the materials used, and the design time. Although some custom orthoses can be economically competitive, more complex applications remain expensive and insufficiently integrated into current reimbursement systems. The prospects for development are numerous, however. The development of biocompatible and sterilisable materials paves the way for devices that can be used directly on patients, whereas improvements in articular volumetric segmentation techniques, particularly in rheumatoid arthritis, could refine the personalisation of devices. Additionally, the emergence of modelling tools from scanners integrated into smartphones could facilitate access to custom orthoses, subject to rigorous technical and clinical validations.

More innovative applications, such as the design of custom implantable prostheses, particularly in advanced rhizarthrosis, or the 3D printing of medications, open prospects for individualised care. The possibility of more precise dosing and a gradual tapering [20], for example, in inflammatory diseases, is particularly attractive but raises questions of safety, drug interactions, and regulatory frameworks.

We will not delve here into the vast potential of 3D printing in fundamental research, but it is immense. This technology enables the production of small batches to test various shapes (robotic arms, orthoses, damaged machine parts, etc.), different materials (eg, bioresorbable or durable, soft or hard, radiolucent or radiopaque, flexible or rigid), and combinations of materials (layered or applied, such as for drug coating or tissue mimicking). It also allows for the exploration of their biocompatibility, among other possibilities. In conclusion, although 3D printing holds significant promise for rheumatology and the broader medical field, its integration into clinical practice depends on stronger evidence, cost-effectiveness, and sustainable practices. Its development could be advanced through academic initiatives—such as individual centres or networks of academic institutions producing 3D-printed devices—as well as through collaborations with 3D printing firms to conduct formal clinical trials. However, this progress will require strengthening the evidence base, performing rigorous medico-economic and environmental assessments, and establishing robust regulatory frameworks to ensure a reasoned and sustainable adoption.

## Editor disclosure

The peer review process did not involve Editorial Board Member Alain Saraux, and the editorial decision-making was led by editors who were not involved in the creation of this manuscript.

## CRediT authorship contribution statement

**Samuel Guigo:** Writing – review & editing, Visualization, Validation, Supervision, Resources, Formal analysis, Data curation, Conceptualization. **Sophie Débonnaire:** Writing – review & editing, Writing – original draft, Visualization, Validation, Methodology, Investigation, Data curation, Conceptualization. **Zoé Rapin:** Writing – review & editing, Writing – original draft, Visualization, Validation, Methodology, Investigation, Data curation, Conceptualization. **Alain Saraux:** Writing – review & editing, Writing – original draft, Visualization, Validation, Supervision, Methodology, Investigation, Formal analysis, Data curation, Conceptualization.
